# Assessing exposure to Kilkari: a big data analysis of a large maternal mobile messaging service across 13 states in India

**DOI:** 10.1136/bmjgh-2021-005213

**Published:** 2021-07-26

**Authors:** Jean Juste Harrisson Bashingwa, Diwakar Mohan, Sara Chamberlain, Salil Arora, Jai Mendiratta, Sai Rahul, Vinod Chauhan, Kerry Scott, Neha Shah, Osama Ummer, Rajani Ved, Nicola Mulder, Amnesty Elizabeth LeFevre, Kilkari Impact Evaluation Team, Smisha Agarwal

**Affiliations:** 1 Computational Biology Division, Department of Integrative Biomedical Sciences, Institute of Infectious Disease and Molecular Medicine (IDM), Faculty of Health Sciences, University of Cape Town, Cape Town, South Africa; 2 International Health, Johns Hopkins School of Public Health, Baltimore, MD, USA; 3 BBC Media Action, Delhi, India; 4 Beehyv Software Solutions Pvt Ltd, Hyderabad, India; 5 International Health, Johns Hopkins University Bloomberg School of Public Health, Baltimore, Maryland, USA; 6 International Health, Johns Hopkins Bloomberg School of Public Health, Baltimore, Maryland, USA; 7 Oxford Policy Management-Delhi, Delhi, India; 8 National Health Systems Resource Centre, New Delhi, Delhi, India; 9 School of Public Health and Family Medicine, University of Cape Town, Cape Town, South Africa; 10 International Health, Johns Hopkins Bloomberg School of Public Health, Baltimore, MD, USA

**Keywords:** maternal health, health systems evaluation

## Abstract

The Kilkari programme is being implemented by the Government of India in 13 states. Designed by BBC Media Action and scaled in collaboration with the Ministry of Health and Family Welfare from January 2016, Kilkari had provided mobile health information to over 10 million subscribers by the time BBC Media Action transitioned the service to the government in April 2019. Despite the reach of Kilkari in terms of the absolute number of subscribers, no longitudinal analysis of subscriber exposure to health information content over time has been conducted, which may underpin effectiveness and changes in health outcomes. In this analysis, we draw from call data records to explore exposure to the Kilkari programme in India for the 2018 cohort of subscribers. We start by assessing the timing of the first successful call answered by subscribers on entry to the programme during pregnancy or postpartum, and then assess call volume, delivery, answering and listening rates over time. Findings suggest that over half of subscribers answer their first call after childbirth, with the remaining starting in the pregnancy period. The system handles upwards of 1.2 million calls per day on average. On average, 50% of calls are picked up on the first call attempt, 76% by the third and 99.5% by the ninth call attempt. Among calls picked up, over 48% were listened to for at least 50% of the total content duration and 43% were listened to for at least 75%. This is the first analysis of its kind of a maternal mobile messaging programme at scale in India. Study analyses suggest that multiple call attempts may be required to reach subscribers. However, once answered, subscribers tend to listen the majority of the call—a figure consistent across states, over time, and by health content area.

**Summary box**

- In this analysis, article we have sought to assess exposure to Kilkari—the world’s largest mobile maternal messaging programme—across 13 states in India.
- This is the first study of its kind to assess subscriber exposure to mobile messages of a programme at scale.
- Exposure to health information messages drives impact on health outcomes.
- Improved understanding of subscriber engagement with Kilkari calls is vital to deepening programme reach and impact.

## Introduction

Mobile phones are increasingly being used to deliver health information directly to beneficiaries; particularly in low resource settings where individuals often lack access to high-quality health information. Maternal messaging programmes, which deliver stage-based, time-sensitive health information to pregnant women and/or new mothers, are among the few examples of digital health programmes to have scaled widely in a number of settings. Maternal messaging programmes in Bangladesh,^1^ India,^2 3^ Tanzania^4 5^ and South Africa^6 7^ have all attained over 1 million subscribers each.

Despite the reach of maternal messaging programmes in terms of the absolute number of subscribers, little has been reported about subscriber exposure to content over time, by digital channel—email, short messaging service (SMS); interactive voice response (IVR)—or by health content area.^8^ The lack of reporting is attributed in part to limitations in available data and analytic capabilities both in terms of skill and available computing power. For IVR programmes that send outbound prerecorded voice calls directly to subscribers’ mobile phones, available data include call data records (CDRs), which may capture a range of data elements linked to exposure including delivery status and duration of subscriber listening to individual calls. CDRs can be as large as 100 GB for just a few weeks or months of call data, and, as a result, require skills in data science and the appropriate computing infrastructure support—both of which may be in short supply in the development sector. When assessed over time and across geographic areas, CDRs can provide important insights into user engagement with mobile messages and, ultimately, exposure. Despite their immense potential, few examples of CDR analyses exist in the literature—in effect, limiting understanding of exposure within and across mobile messaging programmes. Process evaluations of MomConnect in South Africa^6^ and the Mobile Technology for Community Health programme in Ghana^9^ are exceptions.

In South Africa, MomConnect—a national SMS programme for new and expectant mothers—captured data on attempts made by target beneficiaries to register for the programme using Unstructured Supplementary Service Data as well as data on the receipt of SMS messages.^6^ In Ghana, the Mobile Technology for Community Health programme, which used IVR calls to deliver content to expectant mothers, captured data on calls answered, as well as user engagement with individual calls, including the duration of listening to content over time and across thematic content areas and geographic areas. Findings suggest that although user engagement with content was high when calls were answered in Ghana (subscribers listened to over 80% of all the calls that they answered), call answer rates were low with less than 25% of scheduled calls being answered by pregnant women.^9^ Moreover, call answer rates decreased over time as more users were subscribed to the programme.^9^ By 6–12 months postpartum, less than 6% of enrolled women had answered at least one call.^9^ Findings from Ghana underscore the need to measure exposure as part of digital health programmes, devise strategies to enhance listening rates and understand the linkage between exposure and health behaviours and outcomes.^9^

**Figure 1:**
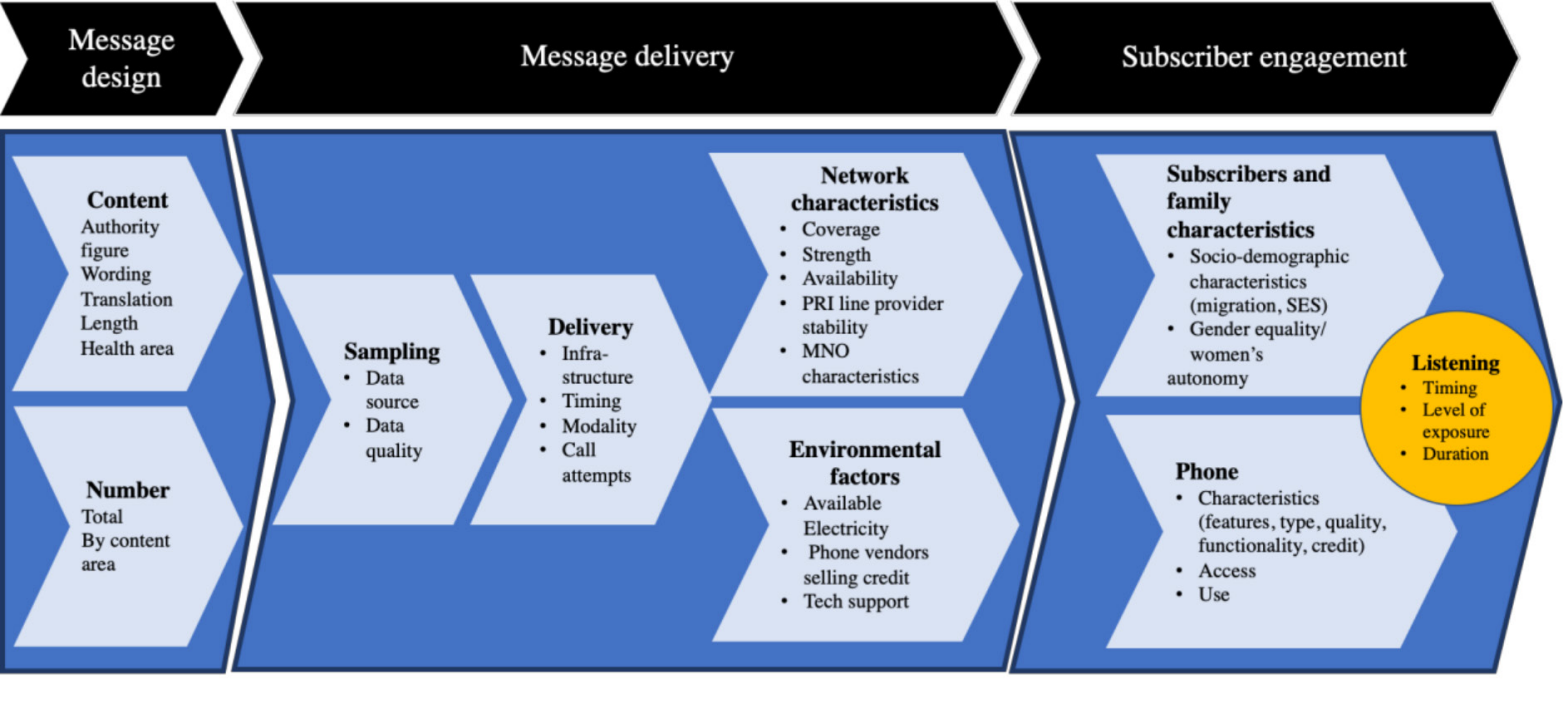
Measuring Kilkari exposure.

Exposure to maternal messaging programmes (email, SMS, IVR, etc) is driven by a range of factors starting with solution design and culminating with subscriber engagement (figure 1). Solution design may vary across programmes and countries and include health focus areas, communication frameworks or curriculums, technical health information, creative approaches to the brand and content creation, technology choices and delivery channels and the duration, frequency and quantity of communication. Content delivery is influenced by the quality of the subscriber data underpinning the programme (mobile phone numbers and the health data required to create a personalised schedule of communications), available infrastructure and communication protocols, including the timing of communications and the number of attempts routinely made to reach subscribers. Factors outside the programme’s control include the characteristics of the mobile phone networks involved in content delivery, the status of the subscriber’s phone and network connection (eg, no battery, no credit, broken device) and the broader technology environment (eg, electricity to charge phones, proximity of top-up vendors, technology support). Both content design and delivery interact with the characteristics of subscribers and their families and social norms that influence women’s access to and use of devices to determine subscriber engagement and, ultimately, listening.

In this analysis, we explore exposure to the Kilkari programme in India for those who subscribed to the service in 2018 (box 1). We start by exploring the timing of first exposure—defined as the first time a subscriber answers a Kilkari call. We then present data on average call answer rates by content and over time and assess the timing of calls answered per day and the number of call attempts required to reach subscribers. Next, we explore listening levels, including the duration of listening overall, by content area and over time. We close by assessing linkages between the timing of the first call answered during pregnancy or postpartum with overall levels of listening observed. This is the first analysis of its kind of a maternal messaging programme at scale. Efforts to quantify the effects of both content delivery and subscriber behaviour are anticipated to provide crucial insights on Kilkari exposure and, in turn, inform the refinement of programmatic design.

Box 1 **Overview of the Kilkari Programme**

**Program description**

Kilkari is an outbound service that makes weekly, stage-based, prerecorded calls about reproductive, maternal, neonatal and child health directly to families’ mobile phones, starting from the second trimester of pregnancy until the child is 1-year old. The timing of messages is based on the pregnant woman’s last menstrual period or the child’s date of birth (if available). BBC Media Action designed and piloted Kilkari in the Indian state of Bihar in 2012–2013 and then redesigned and scaled it in collaboration with the Ministry of Health and Family Welfare between 2015 and 2019.

**Retry algorithm**

In an optimal scenario, subscribers receive and listen to the first call sent out. However, calls might not reach end-user devices or be picked up. Kilkari attempts to call subscribers multiple times as part of a ‘retry algorithm’, which consists of resending the same message up to nine times—three attempts are made the first day, and two attempts for the next 3 days. If the call still does not reach the end-user device or is not picked up, the MOTECH engine that sends out Kilkari messages will move onto the following weekly audio message.

**Deactivation**

Subscribers can self-deactivate from the Kilkari at any time or be manually deactivated for low listenership. Originally, subscribers who listened to <25% of the content on average for 12 weeks were considered low listeners. With new states being added to the programme without a concurrent increase in the system capacity, the definitions, over time, have been made stricter so as not to overload the capacity of the system to make calls. Deactivation of low listeners has been carried out because there was insufficient room in the back-end database to accommodate all the records for all the states, so high listeners were prioritised. At the time of this analysis, subscribers who listened to <25% of the content on every call for 6 weeks were considered low listeners and subsequently deactivated. The timeframe for the calculation of low listenership has varied widely, sometimes 12 weeks, 6 weeks or even occasional suspension of the deactivation process.

## Timing of first exposure: *58% of subscribers received and answered their first call after childbirth*

The Kilkari programme is described in-depth elsewhere^2 3^ and in box 1. In brief, the government’s databases that register and track all pregnancies and births in India share key data with the Kilkari profile database (called MOTECH), including the date of the woman’s last menstrual period or the due date of the child and the mobile number registered with her record. The Kilkari system uses this data to create a schedule of weekly calls for each subscriber, based on the woman’s stage of pregnancy or her child’s age (box 2). Subscribers should receive calls each week for up to 72 weeks (from the second trimester of pregnancy until the child is a year old). The timing of the first call is determined by the data shared by the government’s databases. Whether calls are delivered, answered and listened to depend on the many factors described in in figure 1.

Box 2 **Methods**

**Analytic time horizon**

While programme implementation commenced in 2012, the analytic time horizon was restricted to 1 January to 31 December 2018. This represented the first calendar year during which implementation had occurred for all 13 states.

**Modeling expected messages in 2018:**

Efforts to model the number of expected messages for 2018 assumed a two-step process:

Step 1: the number of weeks between the date of last menstrual period (LMP) and creation date (mother subscription) or weeks between child date of birth and creation date (child subscription) was used to calculate the expected calls as defined in online supplemental table 1.

N=Number of weeks from LMP to creation date

Or N=Number of weeks from child Date of Birth (DOB) to creation date

*For pregnant women (mother pack)*

Expected calls=72 if N≤12 weeks

or expected calls=72–N+12 if N>12 weeks

*After delivery (child pack)*

Expected calls=48 N

Step 2: the number of weeks between the creation date and the 31 December 2018 was used to calculate the expected calls in this specific timeframe.

N1=number of weeks from creation date to the 31 December 2018

Expected calls 2018=expected calls if N1>expected calls

or expected calls 2018=N1 if N1≤expected calls.

10.1136/bmjgh-2021-005213.supp1 Supplementary data

**Figure 2:**
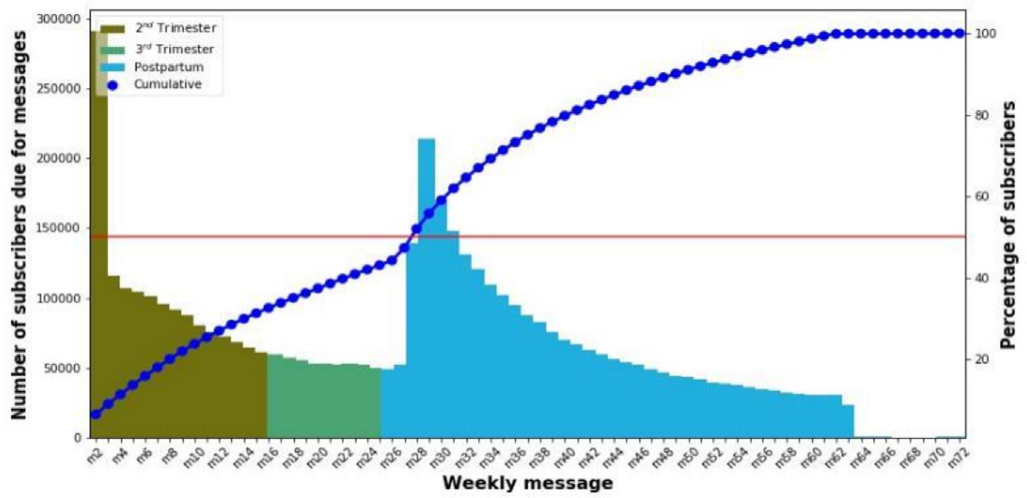
Timing of the first message answered by Kilkari subscribers.

Thirty one per cent of pregnant women who subscribed to Kilkari in 2018 answered their first call before the start of their third trimester, 11% answered during the third trimester and 58% answered during the postpartum period (figure 2). Across geographic areas, the proportion of subscribers answering the first call during the pregnancy period varied widely from state to state, ranging from 1% of subscribers in Rajasthan to 79% in Assam (online supplemental figure 1). This variation results from the timeliness of data collection (pregnancy registration data collected by frontline health workers), and whether this data are first entered into state government databases or directly into national government databases. If it is first entered into state-based databases (eg, in the case of Rajasthan), a delay in the flow of data into the Kilkari database was observed. In order to increase exposure to Kilkari calls during pregnancy, efforts are needed to improve the timeliness of data collection and entry into government databases and the flow of data from the states to the central government.

## Call volume: *1.2–1.4 million calls made daily*

**Figure 3:**
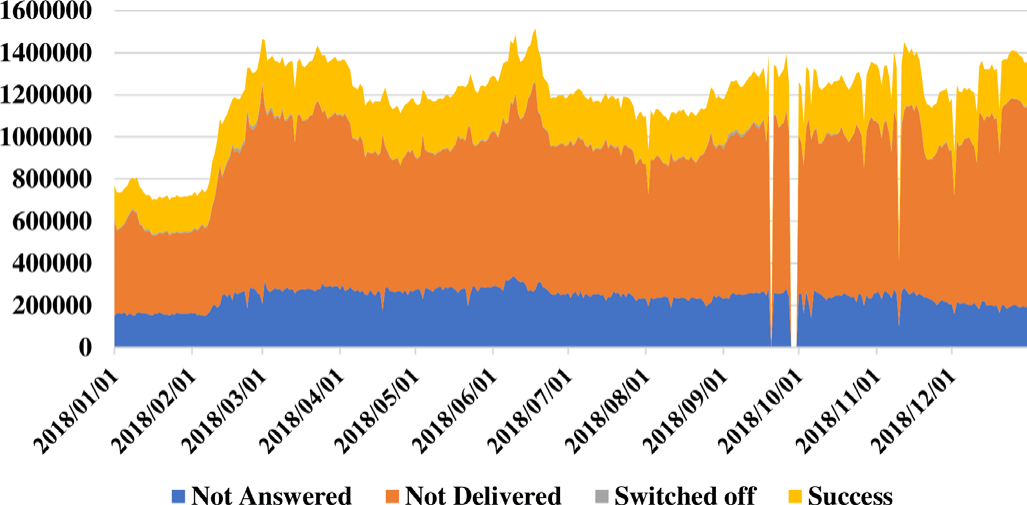
Call volumes across 13 states in India from January to December 2018.

Kilkari made 1.2 to 1.4 million calls per day (including all call attempts) for beneficiaries subscribed in 2018. Calls were made between the hours of 7:00 am and 8:45 pm, which is an extension of the ‘social hours’ when prerecorded calls can be made as per Telecom Regulatory Authority of India regulations (prerecorded calls cannot be made from 9 pm to 9 am), which are considered ‘antisocial hours’. Call volume data suggest that the volume of calls made is consistent from 7 am to 6 pm, tapering slightly thereafter until 8 pm (online supplemental figure 2). Of those calls, 66% were not delivered (did not reach the subscriber’s handset). Seventeen per cent of all calls were not answered (despite reaching the handset) and 17% were successfully answered by someone who listened to at least 1 second or more of the call (figure 3). At the state level, West Bengal had the highest delivery rate (calls reaching the handset) at 42%, while Chhattisgarh had the lowest delivery rate at 26% (online supplemental table 3). Nondelivery could primarily be attributed to reasons beyond the programme’s control, as detailed in figure 1. For example, Chhattisgarh is a primarily rural state with large wilderness areas, limited infrastructure and exceptionally low mobile network coverage. Subscriber reasons for not answering calls could be attributed to a range of factors including subscriber’s being engaged with work or domestic activities, phone ownership and sharing practices, perceptions of the need for health information and awareness of Kilkari. Variations across states might be attributed to state-level variations in these factors, especially mobile network coverage and quality and phone ownership and sharing practices.

## Kilkari message exposure: *75% of Kilkari messages were answered*

**Figure 4:**
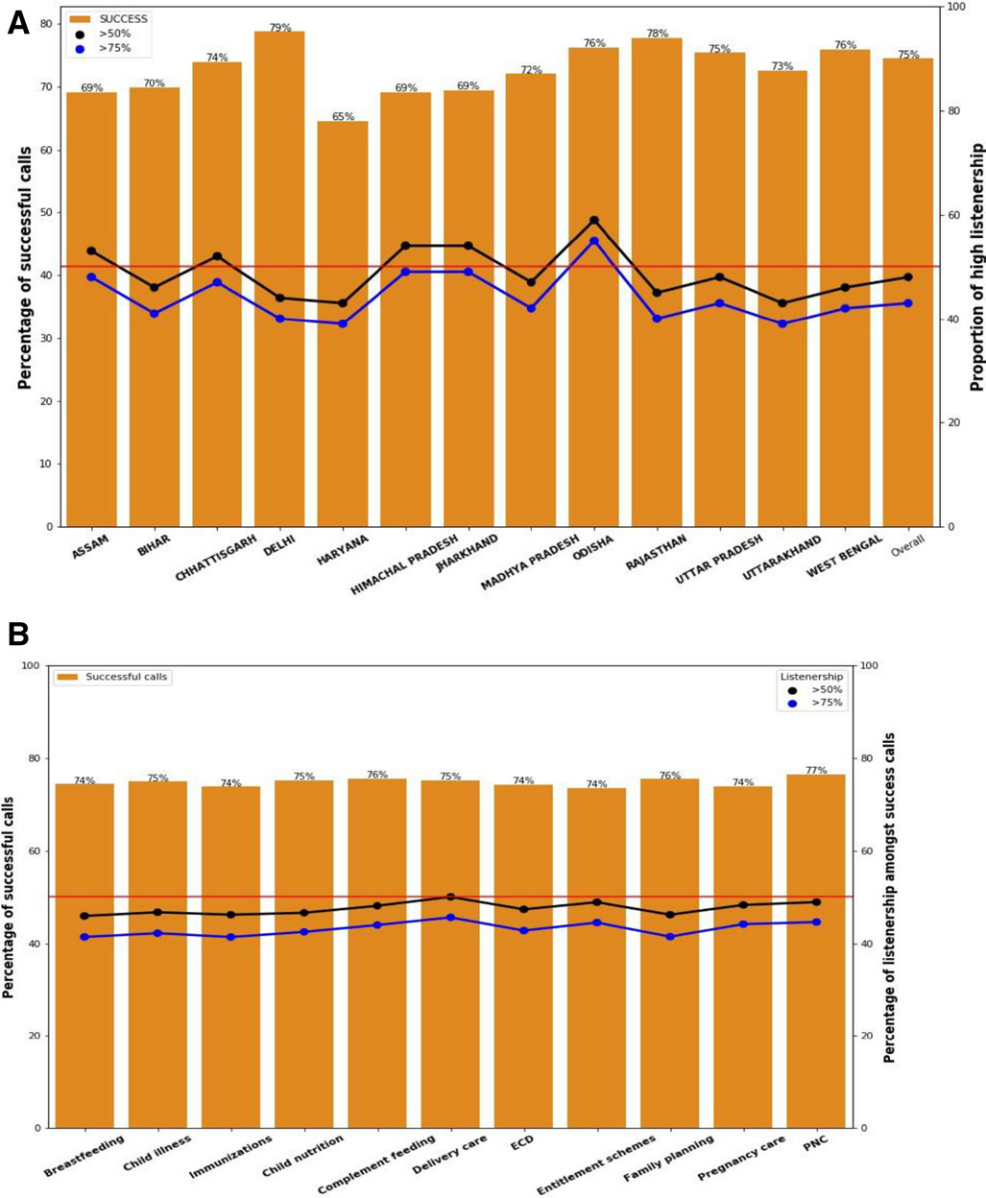
(A) Proportion of successful calls and high listeners by state for 2018. (B) Proportion of successful calls and high listeners by content area for 2018.

Figure 4A shows the proportion of calls that are answered out of all calls made and the proportion of high listenership at listening thresholds of >50% and >75% of content across states. Overall trends are similar across 13 states and suggest that 75% of all scheduled Kilkari calls were delivered and answered, thanks to multiple call attempts/retries (see the section below on the retry algorithm). Trends in successful calls were consistent over time and across message content areas (figure 4B).

## Retry algorithm: *over half of successful calls were answered by the third attempt*

**Figure 5:**
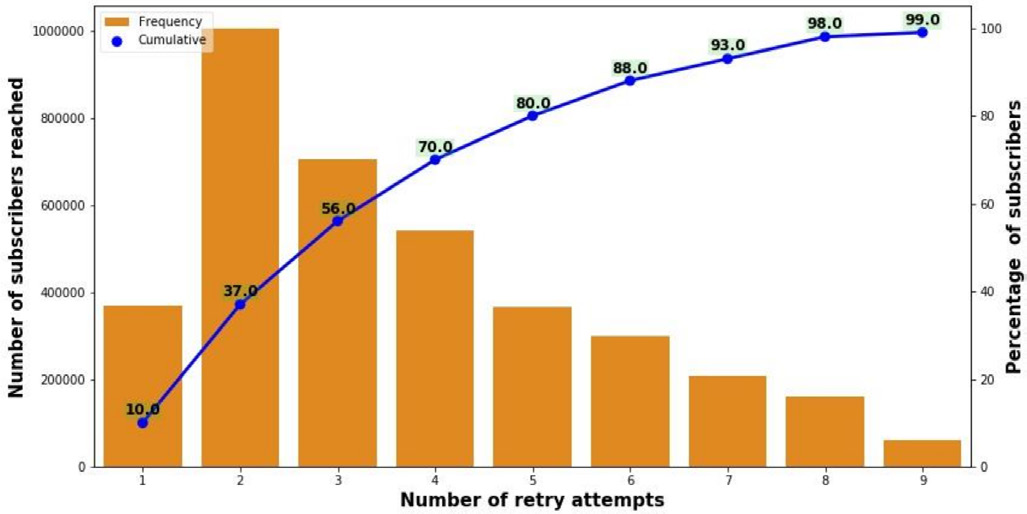
Cumulative number of subscribers who receive and answer a call by the average number of retry attempts.

Kilkari attempts to call subscribers up to nine times each week in an effort to yield a successful call (defined as a call that is answered). On average, 56% of subscribers answered the call by the third call attempt and 99% by the ninth retry attempt (figure 5). When assessed in terms of successful calls (vs calls delivered to the handset and not answered), on average, 50% of calls were answered on the first attempt, 76% by the third and 99.5% by the ninth retry attempt (online supplemental figure 5). Overall findings suggest that the call retry algorithm designed by BBC Media Action is successful in reaching target subscribers and that any reduction below nine retries would reduce the number of successful calls. Additional analysis has revealed that more retries are required to reach the most marginalised subscribers with the lowest levels of mobile network connectivity. The implications of the latter are explored elsewhere.^10^

## Timing of call pick up: *peak times for answering calls are before 10 am and after 6 pm*

**Figure 6:**
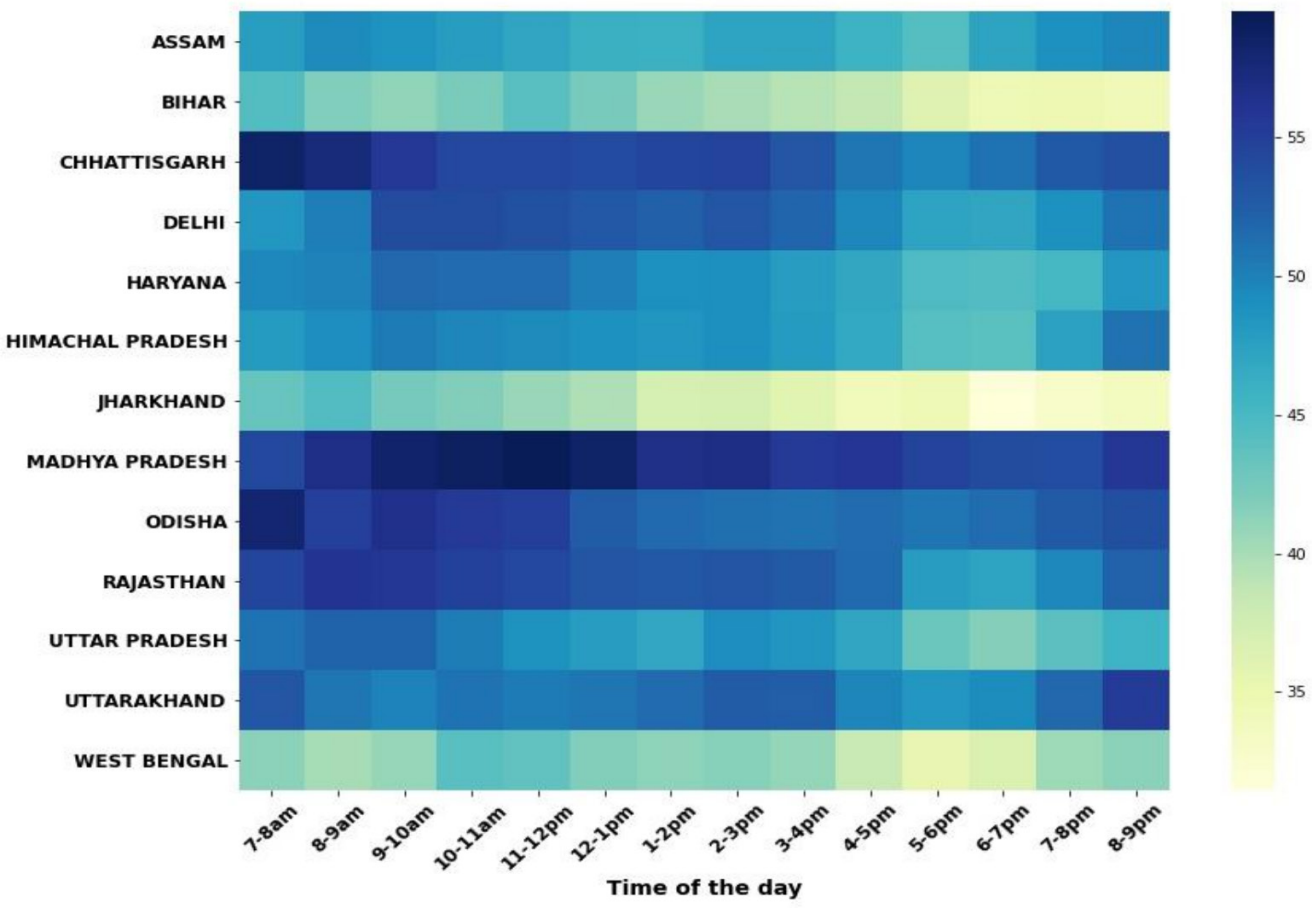
Timing of calls* answered by subscribers across state and by time of day. *The denominator here is restricted to only those calls delivered to the subscriber’s handset. Reasons for not answering could include mobile switched off or the subscriber deciding not to answer.

The proportion of calls answered by subscribers ranged between 50% and 51% in the morning before 10 am to 43%–47% in evening from 6 to 9 pm (figure 6). Peak answering rates varied slightly from state to state, which is a likely indication of differences in the characteristics of subscribers. Bihar, Jharkhand and West Bengal were exceptions; broadly showing lower overall call answer rates throughout the day and particularly after 5 pm.

## Duration of listening: *subscribers listen to more than 50% of the content on nearly half of all calls answered*

**Figure 7:**
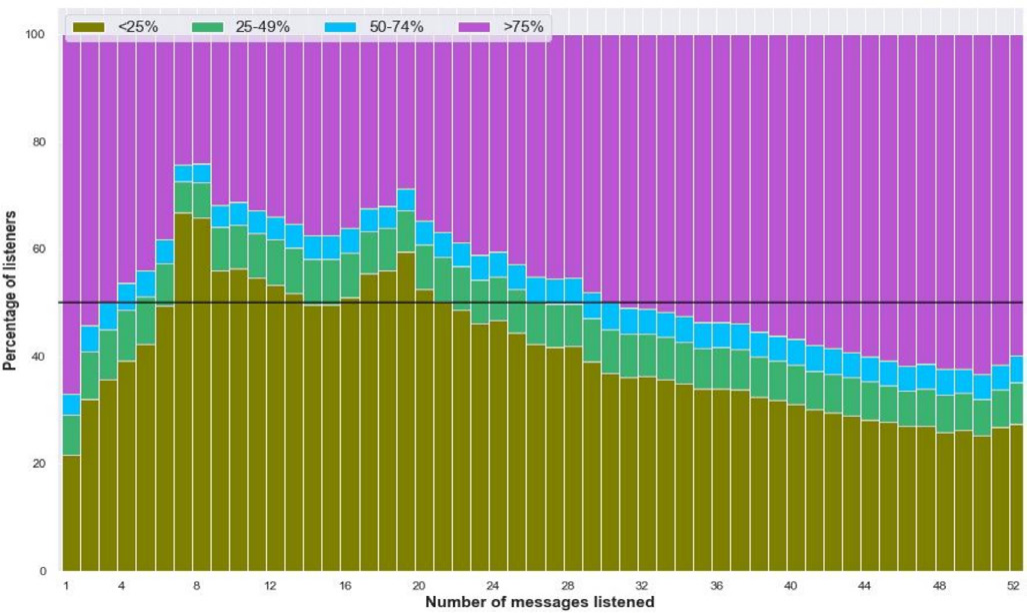
Duration of listening based on the number of messages listened to.

The audio content delivered in each Kilkari call is approximately 2 min in length. Each call introduces a health topic, provides information about the topic and a call to action and then summarises the content of the call again. For practical purposes, listening to the first 50% of each call provides the listener with all the information they need about that health topic. The Kilkari programme, thus, considers listeners to have been ‘exposed’ to content if they have listened to 50% or more of the content, and segments listeners into the following listening categories—0%–25%, 25%–49%, 50%–74%, 75%–100%—based on the average duration of content heard for the calls they answered. For the purposes of this analysis, we adopted the same approach to segmentation. Among successful calls, over 48% were listened to for 50% or more of the total content and 43% were listened to for 75% or more. These findings suggest that when calls are answered, subscribers usually listen to more than 50% of their content. At the individual subscriber level, the percentage of high listeners increases as the aggregate number of messages heard by the subscriber increases (figure 7). This may be a pointer to the level of engagement with Kilkari (those who listen actively to messages listen through the duration of the programme) but could also be the result of the Kilkari programme manually deactivating low listeners to make room for subscribers who wanted to listen to calls.

## Listening levels were similar over time and by content area

High listenership was steady at state level and across different stages of pregnancy and postpartum (online supplemental table 2). Across health domains, high listenership at both >50% and >75% thresholds was also similar, ranging from 46% to 50% (figure 4B). The proportion of messages that were listened to at >50% of the content was slightly higher among women subscribed to Kilkari during their pregnancy (48%) as compared with those subscribed during the postpartum period (45%). At the level of the individual subscriber, the percentage of high listeners increased as the aggregate number of calls that they listened to increase (figure 7). This finding points to loyal subscribers listening to more content but could also be the result of the Kilkari programme manually deactivating low listeners to make room for subscribers who wanted to listen to calls.

## Conclusions

In this analysis article, we have sought to assess exposure to Kilkari—the world’s largest mobile maternal messaging programme—across 13 states in India. Exposure to mobile health messaging programmes underpins programme effectiveness in improving knowledge, changing practices and generating demand for public health services. Yet, few programmes have analysed CDRs and other available data sets to explore the implications for mobile solution design and implementation. Using big data analytics, we analysed CDR to gain valuable insights into call delivery, answer rates and content listening habits to describe and understand exposure patterns. Overall, the majority of subscribers received and answered their first call after the birth of the child. Overall, 75% of all scheduled Kilkari were answered. The programme’s ‘retry’ algorithm, which attempts to call subscribers up to 9 times, to yield one answered call is integral to reaching large numbers of subscribers. Subscribers listened to more than 50% of the content on all calls answered; a rate which was consistent over time. Similar listening patterns were observed across states and health content areas. The deactivation of low listeners may overestimate observed estimates of listening. This is the first analysis of its kind to explore call delivery, answer rates and listening habits and patterns in exposure.

## Data Availability

Data may be obtained from a third party and are not publicly available.
