# Supplementary material for: Assessing exposure to Kilkari: a big data analysis of a large maternal mobile messaging service across 13 states in India
Source: BMJ Glob Health. 2021 Jul 26;6(Suppl 4):e005213. doi: 10.1136/bmjgh-2021-005213 (PMC8327807; doi:10.1136/bmjgh-2021-005213)

Supplementary tables and figures

Supplementary Table 1. Terminology

Term	Description
Successful call	Call was answered
Switched off	Call reached mobile device but mobile device was switched off
Not answered	Call reached mobile device but was not answered
Not delivered	Call did not reach mobile. Reasons may include mobile was out of network range; network signal was down in that area; or network congestion prevented call from getting through.
Duration of message played	Duration of that an audio file was played to a subscriber during a Kilkari call
Subscriber or beneficiary	Unique MCTS / RCH ID number linked to a phone number subscribed to Kilkari in MOTECH
Subscriber reached	Subscribers who answer at least one of the first six weekly calls made by the program
Creation date	Date where a new subscription is created into Motech (for women with first pregnancy/ first birth). It is also the date where Motech get an update for new pregnancy or childbirth for women already subscribed into Motech.
Expected calls	Total number of calls subscribers are supposed to receive on their registration to Kilkari based on last menstrual period or child date of birth.
Calls sent out (pushed out)	Total number of calls in CDR per subscriber, considering one record per weekly message.
Duration of content	Percentage of the total duration of a message that was listened to by a subscriber answering the call
High listenership (>50%)	Listening to at least 50% of the duration of content of one call
Calls delivered	Calls successfully pushed out i.e. calls recorded as successful, not answered or phone switched off

Supplementary Table 2: High listenership amongst successful calls over stage of pregnancy/ postpartum

State	0-12 weeks	12-28 weeks	28-42 weeks	>42 weeks
	n (%)	n (%)	n (%)	n (%)
Assam	27,104 (50%)	1,185,679 (50%)	849,687 (50%)	637,803 (50%)
Bihar	54,652 (40%)	211,502 (50%)	181,548 (50%)	156,306 (50%)
Chhattisgarh	18,192 (50%)	193,400 (50%)	180,381 (50%)	213,885 (50%)
Delhi	13,782 (40%)	399,565 (40%)	368,981 (40%)	534,121 (40%)
Haryana	24,671 (40%)	223,675 (40%)	192,959 (40%)	257,288 (40%)
Himachal Pradesh	3,465 (50%)	150,794 (50%)	134,026 (50%)	219,015 (60%)
Jharkhand	395 (60%)	9,933 (60%)	19,967 (50%)	35,595 (50%)
Madhya Pradesh	54,124 (50%)	393,102 (50%)	491,155 (50%)	819,944 (50%)
Odisha	9,103 (60%)	514,574 (60%)	558,374 (60%)	877,134 (60%)
Rajasthan	378,965 (50%)	432,911 (50%)	483,352 (50%)	2,089,897 (40%)
Uttar Pradesh	184,957 (50%)	1,032,309 (50%)	1,124,243 (50%)	1,485,631 (50%)
Uttarakhand	2,603 (50%)	78,748 (40%)	104,757 (40%)	166,990 (40%)
West Bengal	77,322 (50%)	1,283,106 (50%)	1,484,957 (50%)	5,493,896 (50%)

Supplementary Table 3: Delivery rates (percentages)

State	Delivery rates
Assam	26.8%
Bihar	35.0%
Chhattisgarh	26.3%
Delhi	40.5%
Haryana	27.9%
Himachal Pradesh	31.8%
Jharkhand	36.7%
Madhya Pradesh	27.3%
Odisha	27.4%
Rajasthan	38.0%
Uttar Pradesh	38.2%
Uttarakhand	30.4%
West Bengal	41.6%

Supplementary Figure 1. The distribution of the first message received by subscribers to Kilkari across states

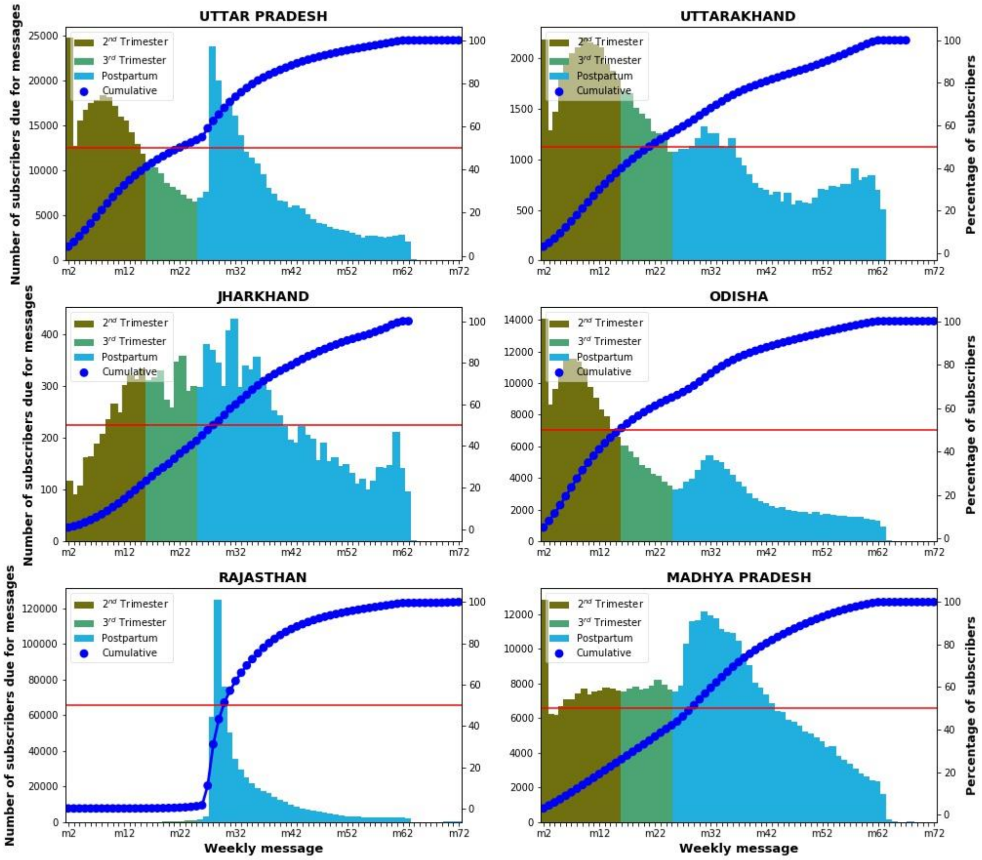

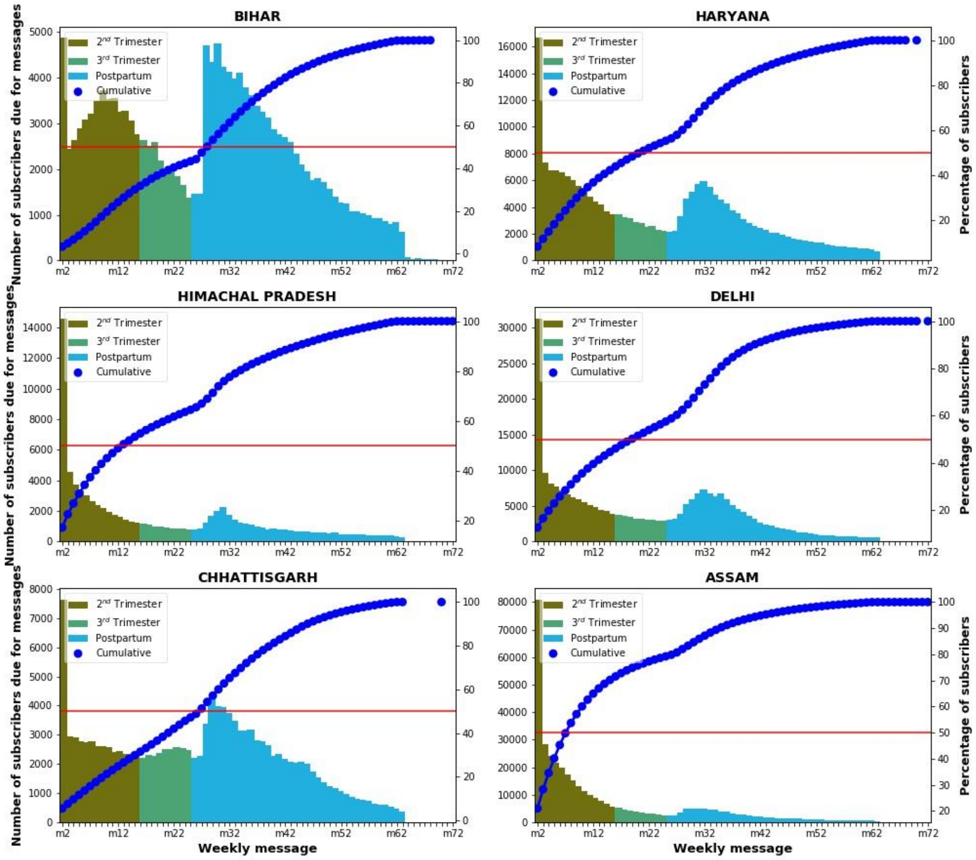
BIHAR
HARYANA
HIMACHAL PRADESH
DELHI
CHHATTISGARH
ASSAM
2nd Trimester
3rd Trimester
Postpartum
Cumulative
Number of subscribers due for messages
Percentage of subscribers
Weekly message

Supplementary Figure 2. Distribution of call volume by time of day

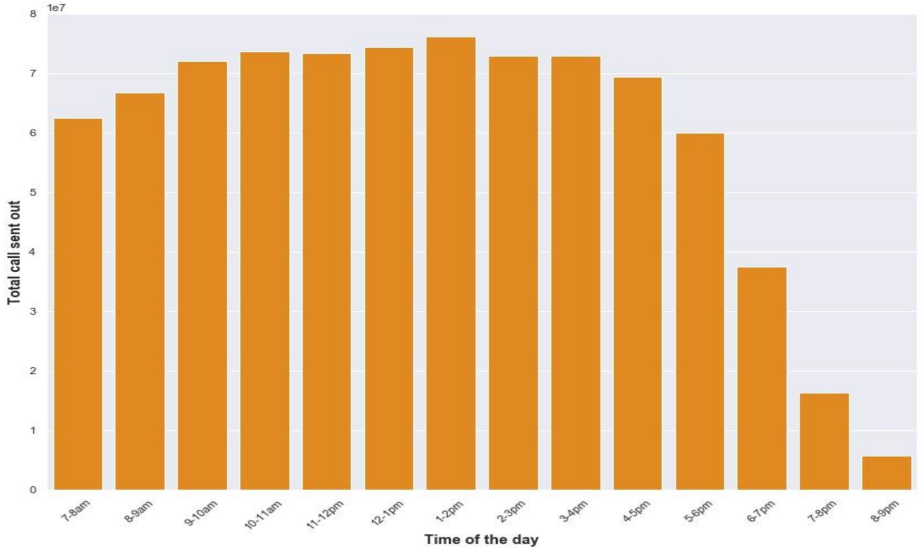

Supplementary Figure 3. Proportion of messages sent out by Motech out of those expected in 2018 across 13 states

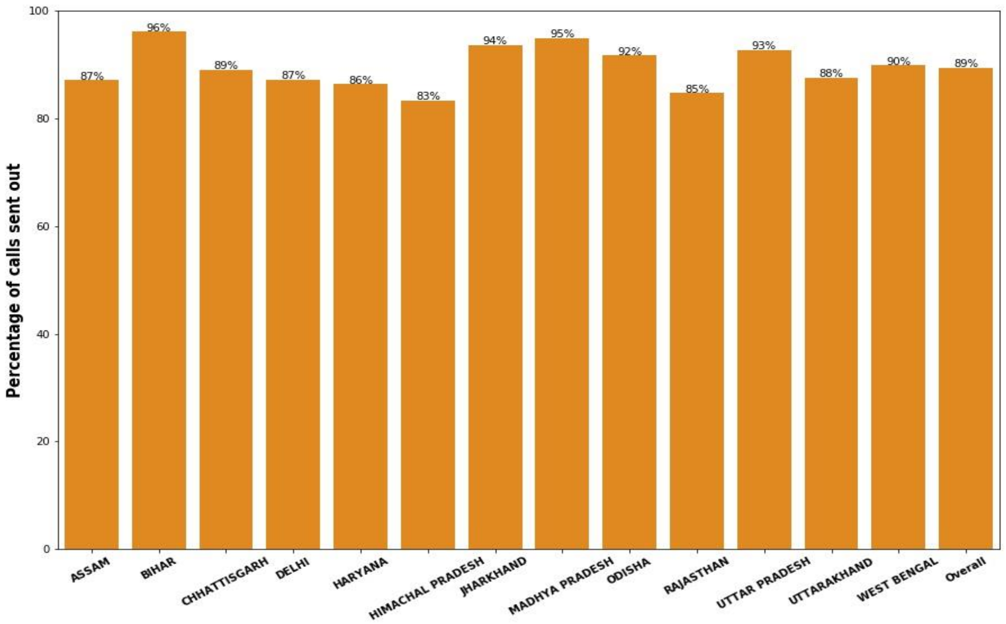

Supplementary Figure 4. Message delivery rates over pregnancy/ postpartum period across states for 2018

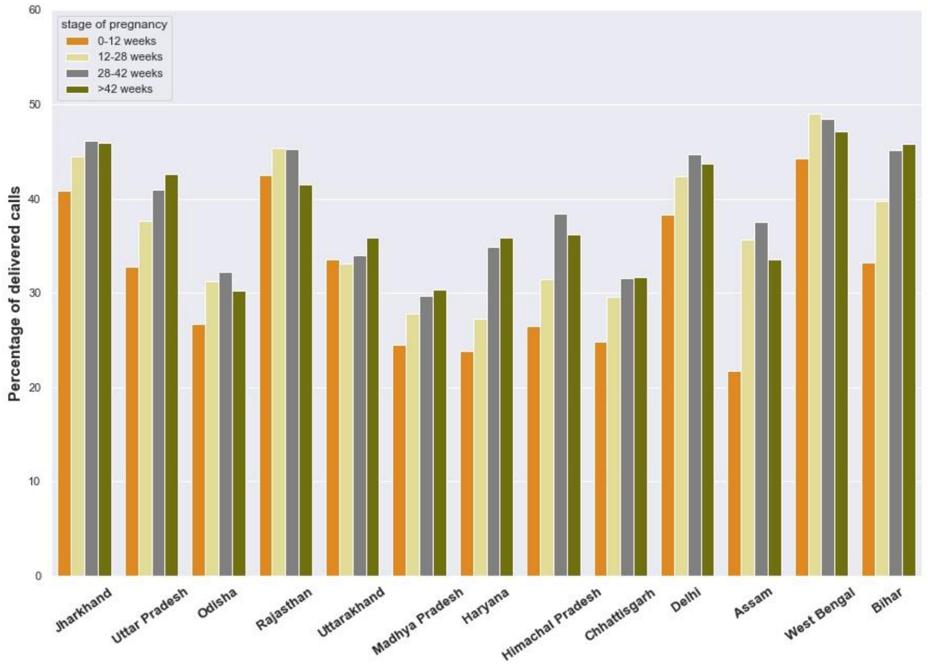

Supplementary Figure 5. The average call attempts (retries) to successfully reach an end-user's device and have a subscriber pick up the call*

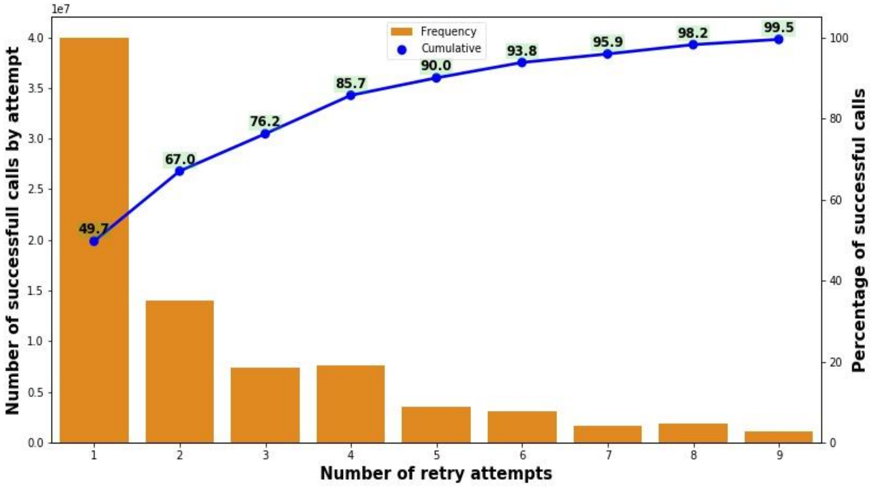

*The call is classified by as 'successful' delivery and thus even if it's picked up and immediately hangs up, it's considered a successful call delivery.

Supplementary Figure 6: Survival curves for women enrolled during pregnancy or postpartum period.

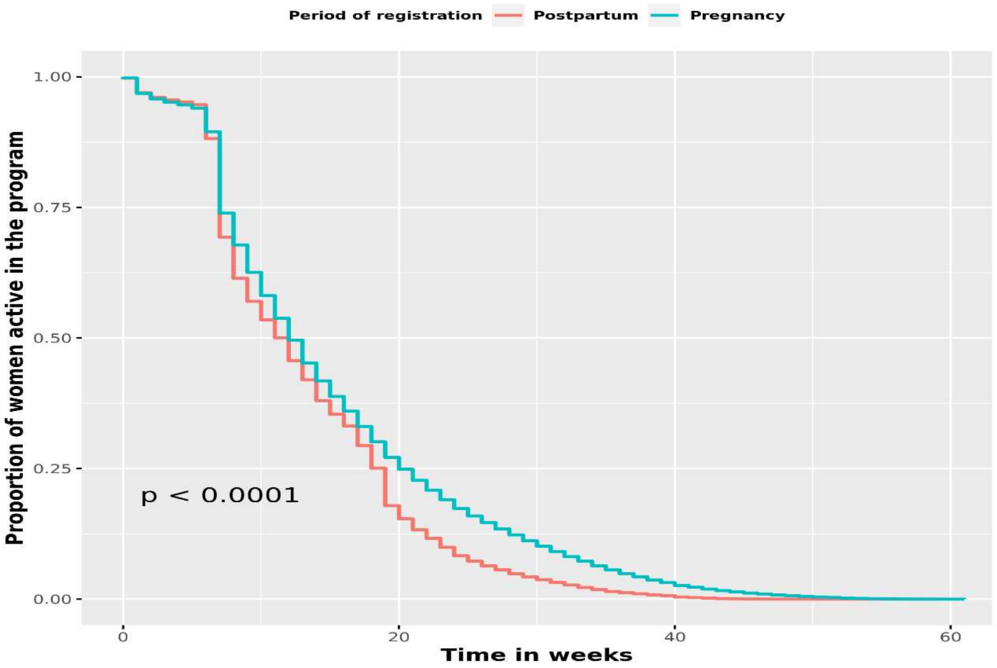

Supplement: Supplementary data [file bmjgh-2021-005213supp001.pdf]
